# Environmental exposure to BDE47 is associated with increased diabetes prevalence: Evidence from community-based case-control studies and an animal experiment

**DOI:** 10.1038/srep27854

**Published:** 2016-06-13

**Authors:** Zhan Zhang, Shushu Li, Lu Liu, Li Wang, Xue Xiao, Zhenzhen Sun, Xichen Wang, Chao Wang, Meilin Wang, Lei Li, Qiujin Xu, Weimin Gao, Shou-Lin Wang

**Affiliations:** 1 Key Lab of Modern Toxicology of Ministry of Education, School of Public Health, Nanjing Medical University, 101 Longmian Avenue, Nanjing 211166, P. R. China; 2 State Key Lab of Reproductive Medicine, Institute of Toxicology, Nanjing Medical University, 140 Hanzhong Rd., Nanjing 210029, P. R. China; 3 Lake Research Center, Chinese Research Academy of Environmental Sciences, Beijing Anwai Beiyuan, Beijing 100012, P. R. China; 4 Department of Environmental Toxicology, The Institute of Environmental and Human Health, Texas Tech University, 1207 Gilbert Drive, Lubbock, TX 79416, U.S.A

## Abstract

Brominated flame retardants exposure has been associated with increasing trends of diabetes and metabolic disease. Thus, the purpose of this study was to provide evidence of polybrominated diphenyl ethers (PBDEs) exposure in relation to diabetes prevalence and to reveal the potential underlying mechanism in epidemiological and animal studies. All the participants received a questionnaire, health examination, and the detection of 7 PBDE congeners in serum in two independent community-based studies from 2011 to 2012 in China. Male rats were exposed to 2,2’4,4’-tetrabromodiphenyl ether (BDE47) for 8 weeks to explore its effects on glucose homeostasis and potential mechanisms using high-throughput genomic analysis. Among the 7 congeners, BDE47 showed significant high detection rate and concentration in cases in Study I and Study II. Every tertile of BDE47 exposure significantly increased the risk of diabetes prevalence in Study I (*P*_trend_ = 0.001) and Study II (*P*_trend_ < 0.001). Additionally, BDE47 treatments induced hyperglycemia in rats. Furthermore, gene microarray analysis showed that diabetes pathway and three gene ontology terms involved in glucose transport were enriched. The results indicated that environmental exposure to BDE47 was associated with increased diabetes prevalence. However, further prospective and mechanistic studies are needed to the causation of diabetes in relation to BDE47.

Polybrominated diphenyl ethers (PBDEs) are a class of brominated flame retardants that are used as additives in polymers for electric and electronic goods and in textiles^1^. Global levels of PBDEs have been detected in air, soil, water, and biota ranging from invertebrates to human, and the levels have increased in the past 30 years^2^,^3^. The source of PBDEs in humans are from external exposure routes (e.g. dust, diet, and air) and the resulting internal exposure to PBDEs (e.g. breast milk and blood)^4^. 2,2′4,4′-tetrabromodiphenyl ether (BDE47) is the dominant PBDE congener in humans, wildlife, and the environment. In animals, BDE47 has been identified as a developmental, reproductive, and neurological toxicant, and a disrupter of multiple endocrine systems^5^,^6^.

With rapid economic growth and associated industrialization, urbanization, and lifestyle changes such as increased high-calorie, high-fat, high-sugar, and high-sodium diets and decreased physical activity, prediabetes and diabetes have reached epidemic proportions in Chinese population^7^. Recently, the overall prevalence of diabetes was estimated to be 11.6% in the Chinese adult population; men might be more susceptible to diabetes than women^8^. Whereas type 1 diabetes (T1D) is largely thought to be of an autoimmune origin, type 2 diabetes (T2D) is mainly associated with obesity and metabolic syndrome though T2D can occur independently of overweight or obesity. Although causal relationships between genetic factors and T2D have been eagerly sought^9^, the data from genome-wide association studies have shown that genetic variants might explain statistically only about 10% of the phenotypic variability^10^. Thus, it has recently been suggested that environmental pollutants should be additional risk factors in diabetes development^11^.

Accumulating epidemiological evidence has demonstrated that exposure to PBDEs might be potentially associated with the risk of diabetes^12^. Our previous study had shown that exposure to BDE209 could induce hyperglycemia in male rats^13^. In addition, BDE153 showed an inverted U-shaped association with serum glucose concentration in a human study^14^. BDE47 could cause disturbance in energy metabolism, and more than 1 μg/L BDE47 could induce higher levels of glucose and ATP in earthworm Eisenia fetida^15^. In previous human studies, including male and female, BDE47 tended to be positively associated with the prevalence of diabetes, but did not reach statistical significance^14^,^16^. Still, the association of BDE47 with diabetes, and the specific underlying mechanism remains unclear.

The purpose of the study was to provide epidemiological evidence of the association between environmental exposure to PBDEs and diabetes, and try to reveal the specific underlying mechanism in an animal model. We adopted two independent community-based case-control studies. In study I, we explored the relationship between PBDEs exposure and diabetes using a 245 paired case-control, which was validated further using an independent 565 paired case-control in study II. In addition, we examined the effects of BDE47 on the onset of diabetes in animals, and conducted the potential mechanism using gene microarray and related bioinformatics analysis. The present study will be helpful to provide better understanding of PBDEs in relation to diabetes.

## Results

### Association between environmental exposure to BDE47 and human diabetes

The prevalence of diabetes was 9.02% (9.56% in men *vs* 8.67% in women) and 9.10% (9.37% in men *vs* 8.92% in women) in crude study I and crude study II, respectively. Men were more susceptible to diabetes than women (Fig. S1). As shown in Table 1, the distribution profile of typical characteristics in cases and controls was similar among the two studies. Family history of diabetes in cases was more frequent than controls in Study I (7.3% *vs* 2.9%) and Study II (5.8% *vs* 1.6%). Additionally, cases were significantly more likely than controls to have hypercholesterolemia, hypertriglyceridemia, dyslipidemia, obesity as well as central obesity in the two studies. Cases also had more frequently reported low HDLC, high LDLC, and hyperuricemia. Further, family history of diabetes (study II), hypertension, dyslipidemia, obesity, and central obesity were shown to be the independent risk factors for diabetes by multivariate logistic regression analysis (Table 2).

All 7 PBDE congeners were detected in cases and controls, BDE28 and BDE47 occupied the top 2 detection rate with more than 50%. Although BDE28 showed a higher detection rate in control group in study I, there was no significant difference in the median serum concentration (study I and study II) and the detected rate (study II) between case and control groups. However, both detection rate and median serum concentration of BDE47 was significantly higher in cases than those in the control, which consistently showed similar results in study I and study II (Table 3). After adjusting for hypertension, dyslipidemia, central obesity, and obesity, there seemed to be a positive relationship between BDE47 and diabetes, but no statistically significant difference (*P* = 0.068). There was a significantly positive association among every tertile of BDE47 with diabetes prevalence in the two studies. The adjusted odds ratios (ORs) in study I were 1.13 and 2.10 in the 1^st^ and 2^nd^ tertiles, respectively (*P*_trend_ = 0.009). Similarly, the adjusted ORs in study II were 1.15 and 1.68 in the 1^st^ and 2^nd^ tertiles, respectively (*P*_trend_ < 0.001) (Table 4).

### Effects of BDE47 on glucose homeostasis in rats

**Figure 1:**
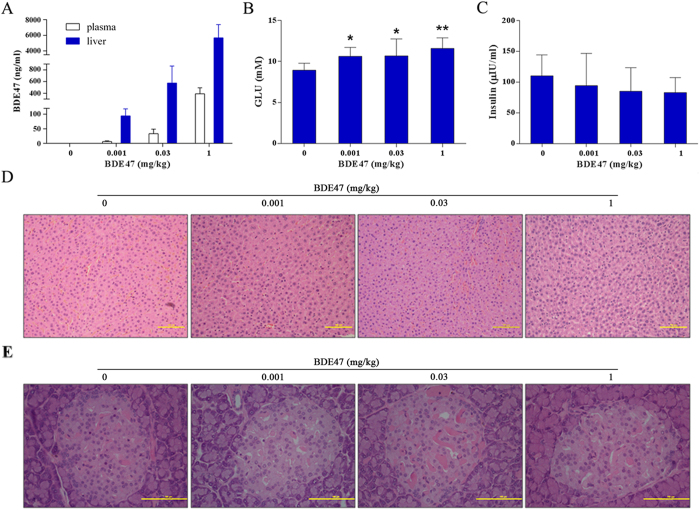
Effects of BDE47 on rat glucose homeostasis and morphological changes in liver and pancreas. (**A**) Concentrations of BDE47 in rat plasma and liver, (**B**) fasting glucose and (**C**) insulin were determined after 8 weeks of BDE47 exposure. The results are expressed as the mean ± SD of 10 rats in each group. *P < 0.05, **P < 0.01, compared with the control group. (**D**) Morphological changes in livers from BDE47-treated rats and controls. Scattered microvesicular steatosis was observed in the liver sections of rats treated with 1 mg/Kg BDE47. (**E**) Morphological observations in pancreas in BDE47-treated rats and controls. The liver and pancreas tissues were stained with hematoxylin and eosin (H&E), and their morphological changes were assessed by an optical microscopy, bar = 100 μm.

The concentration of BDE47 in plasma (3.17, 33.50, and 391.63 ng/mL) and liver (64.27, 573.61, and 5678.95 ng/g) increased dose-dependently (Fig. 1A). Correspondingly, treatment of BDE47 dose-dependently increased the high fasting glucose in rats (Fig. 1B). Although insulin decreased in BDE47 treatment groups, it was not statistically significant in comparison with the control group (Fig. 1C). In addition, scattered microvesicular steatosis was observed in the liver sections of rats treated with 1 mg/Kg BDE47 (Fig. 1D), but no histopathological changes were observed in the pancreas in BDE47 treatment groups (Fig. 1E). Also, no differences in serum ALT, BUN, CHOL, or TG were observed between BDE47 treatment and the control (Fig. S2).

### Potential mechanisms of BDE47 to the onset of diabetes in rats

**Figure 2:**
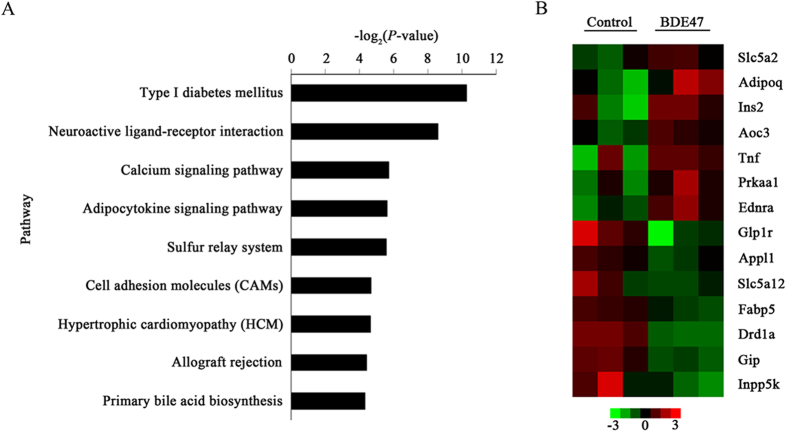
Top-ranking canonical pathways and involved genes in the liver of rat treated with BDE47. (**A**) The top ranking canonical pathways identified by KEGG are listed according to *P*-values. The type I diabetes (T1D) pathway was significantly enriched (*p* < 0.05). *P*-value, probability that the association between the 2-fold differentially expressed genes and the canonical pathways was accounted by chance only. (**B**) Heatmap representation of the selected genes involved in the T1D pathway, glucose transport, regulation of glucose transport, and regulation of glucose import. Gene expression is shown with pseudocolor scale (−3 to 3), with red denoting high gene expression levels and green denoting low gene expression levels of genes.

Compared with the control, 1049 genes were above 2-fold differentially expressed (*P *< 0.05) in BDE47 treated rats: 478 under expressed and 571 over expressed (Table S1). Gene ontology (GO) analysis showed that 54 GO terms were enriched (p < 0.01, FDR < 0.01) (Table S2), and 9 canonical pathways were significantly enriched, among which T1D pathways listed as the top one (Fig. 2A). The differentially expressed genes were involved in 3 target GO terms (glucose transport, positive regulation of glucose transport, and regulation of glucose import) and T1D pathways such as *Tnf*, *Ins2*, *Adipoq*, and *Ednra* (Fig. 2B), which were validated by qRT-PCR (Table S3).

**Figure 3:**
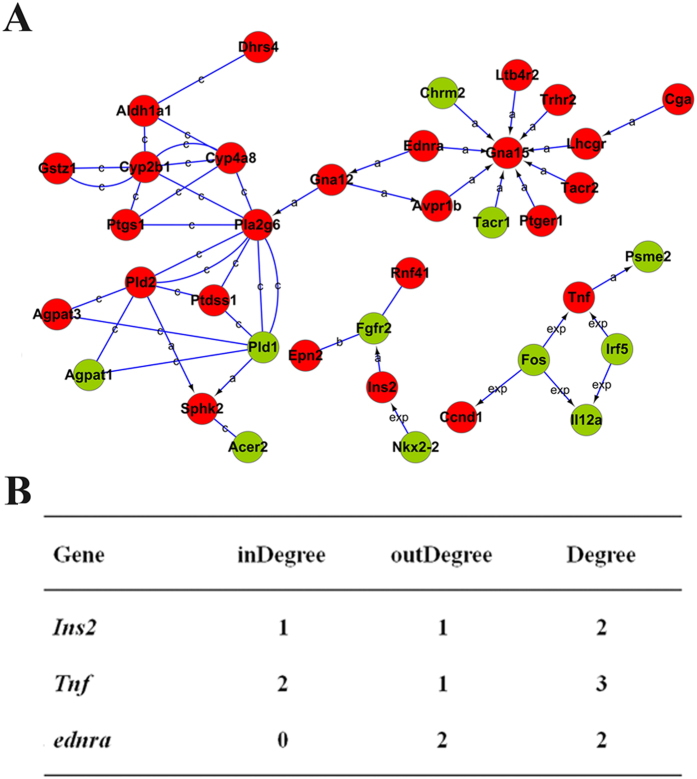
Interactions and functional network identified by KEGG. (**A**) The KEGG database was used to build the network of genes involved in T1D according to the relationship among the differentially expressed genes between BDE47 treatment and control groups. Genes in red and green indicate over- and under-expressed genes, respectively. a, activation; b, binding; c, compound; exp, expression. P-value, probability that the association between the 2-fold differentially expressed genes and the canonical pathway can be accounted for by chance only. (**B**) The degree of involved genes in the gene-act-network. Degree centrality is defined as the link numbers that one node has to the other. InDegree, the number of genes acts on the specific gene; OutDegree, the number of genes is acted on by the specific gene. In the network analysis, degree centrality is the most simplest and important measure of the centrality of a gene within a network that determines the relative importance.

**Figure 4:**
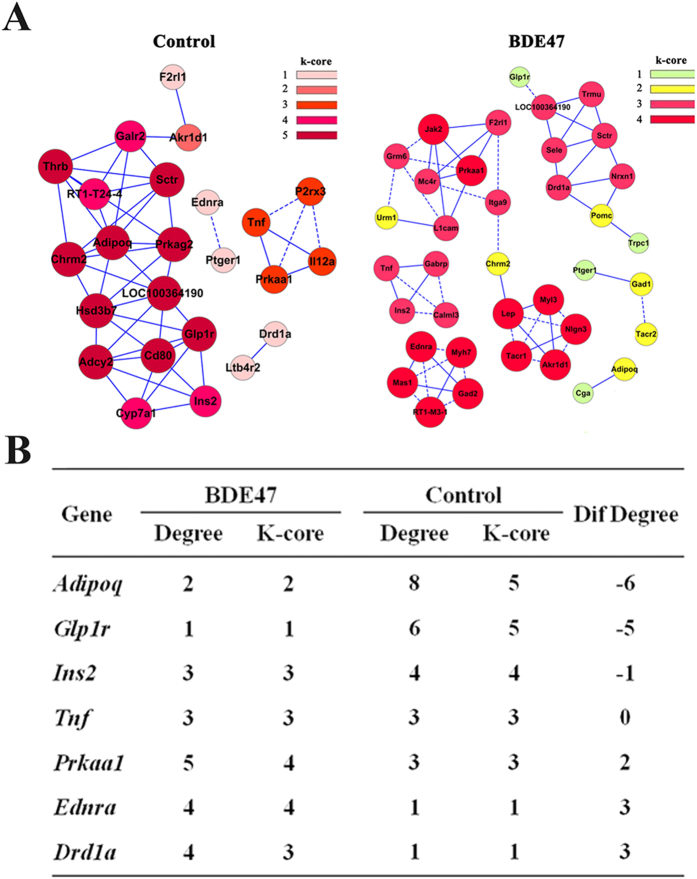
Co-expression network of differently expressed genes in BDE47-treated and control rats. (**A**) Co-expression network of differentially expressed genes in control and BDE47-treated rats. The differential genes that populated in the pathways’ category were selected to build gene co-expression networks according to the normalized signal intensity of specific expression genes. For each pair of genes, we calculate the pearson correlation and choose the significant correlation pairs to construct the network. A k-core of a network is a subnetwork in which all nodes are connected to at least k other genes in the subnetwork. The greater the value of the k-core, the stronger differentially expressed genes co-expressed. Solid line, positively correlated; dashed line, negatively correlated. (**B**) Intersection of differentially expressed genes between BDE47-treated and control rats. Degree centrality is defined as the link numbers that one node has to the other. In the network analysis, degree centrality is the most simplest and important measure of the centrality of a gene within a network that determines the relative importance. While considering different networks, core regulatory factors were determined by the degree differences (Dif gene) between BDE47-treated and control rats.

In the network of gene-gene interaction, *Tnf*, *Ins2*, and *Ednra* were involved in the previously mentioned 3 target GO terms and T1D pathways (Fig. 3A), among which *Tnf* might be the most important due to its strongest degree centrality (Fig. 3B). Further, six networks were identified in the BDE47 treatment group using differentially expressed genes populated in the pathways’ category. Likewise, four networks were identified in the control group (Fig. 4A). To locate core regulatory genes involved in 3 target GO terms and T1D pathways, core regulatory factors were determined by the degree differences between the control and BDE47 treatment groups. As shown in Fig. 4B, *Adipoq* owned the biggest degree of differences followed by *Glp1r*, *Ednra*, and *Drd1a*.

## Discussion

Our study firstly explored the potential relationship between the exposure to BDE47 and diabetes development using both epidemiological and experimental strategies. Results from the pairwise case-control study demonstrated that environmental exposure to BDE47 was positively associated with human diabetes prevalence, which was strengthened by the animal experiment results showing that BDE47 could significantly increase high fasting glucose in rats and enrich T1D pathways with the involvement of some related genes, such as *Tnf*, *Ins2*, *Adipoq*, and *Ednra*. These results indicated that BDE47 might induce the disorder of glycometabolism and potentially result in the onset of diabetes.

The overall prevalence of diabetes was estimated to be 11.6% in the Chinese adult population^8^, most of the participants in this study were rural residents that presented a little low prevalence of diabetes in Study I (9.02%) and Study II (9.10%). Commonly, both metabolic syndrome and family history of diabetes were reported as risk factors of T2D^17^. Family history of diabetes may reflect both genetic susceptibility and exposure to environmental factors that are shared within the family and associated significantly with the presence of diabetes^18^. Consistently, hypertension, dyslipidemia, obesity and central obesity, and family history of diabetes were shown as independent risk factors for diabetes in the present study.

Recently, exposure to polychlorinated biphenyls has emerged as a potential contributor to the pandemic of T2D^19^,^20^. Due to similar structure, PBDEs could induce dyslipidemia which may be involved in the pathogenesis of T2D, and wet-weight concentrations (pg/mL) should have greater validity because lipid concentrations may be intermediate in a causal chain linking PBDEs and T2D^16^. In addition, biological monitoring should be better for the evaluation of environmental exposure because PBDEs are more complicated in the environment but easy to accumulate in the human body. As PBDEs of primary interest (US EPA), 7 congeners were detected in the serum, in which BDE28, BDE47, and BDE183 occupied a higher detectable rate and concentration. Unexpectedly, the detection rate of BDE99 was far lower than other congeners. In addition, the detection rate and concentration of BDE28 was much higher, implying that a different Penta-BDE formulation might be used in the Chinese market^21^. However, only BDE47 showed a significantly positive association with the diabetes prevalence. The result was further confirmed by our animal experiment that BDE47 induced hyperglycemia and impaired glucose homeostasis in male rats even in the lowest dosage (0.001 mg/Kg.d) of BDE47, with about 100-fold of the exposure level in humans in serum concentration (3.17 ng/ml in mean values in rats *vs* 30 pg/ml in median levels in human) (Fig. 1A, Table 3).

Even though it is still unclear what biological mechanism is involved in the association between PBDEs and diabetes, the potential of xenobiotics to disrupt glucose and lipid metabolism in mammals is a commonly accepted theory in toxicology^22^. The biological process categories such as carbohydrate metabolism, electron transport, and lipid, fatty acid, and steroid metabolism were enriched in livers of pups exposed to BDE47^23^. Several GO terms were involved in BDE47-treated rats, such as steroid biosynthetic process and response to retinoic acid. Abnormal metabolism of steroid or retinoic acid might contribute to diabetes^24^,^25^. In addition, another three GO terms involved in glucose transport and T1D pathways was enriched in BDE47-treated rats, in which 7 related genes, such as *Ins*, *Tnf*, and *Adipoq*, were involved. T1D is a serious disorder characterized by destruction of pancreatic β-cells, culminating in absolute insulin deficiency^11^. However, in insulin-resistant states (obesity, prediabetes, and T2D), hepatic production of glucose and lipid synthesis are heightened in concert, implying that insulin deficiency and insulin excess coexists in this setting^24^. TNFα is an important proinflammatory cytokine involved in the pathogenesis of autoimmune T1D^25^,^26^. Adiponectin, which is secreted by the white adipose tissue, is a protein with insulin-sensitivity and anti-atherogenic effects^27^. Variations in *Adipoq* are associated with obesity, T2DM and related phenotypes in several populations^28^. Variation in *Adipoq* locus contributes to variation in body size and serum adiponectin concentrations and may also modify the risk of developing T2DM^29^.

Exposure to BDE47 was associated with T2D in humans while T1D pathways contributed to BDE47 treatment in rats. There seems to be a discrepancy of the type of diabetes between the epidemiological and experimental results in this study. No effects of BDE47 on rat TG or CHOL were observed in the present study. As well known, T2D participants have higher TG and CHOL than controls^30^. No histopathological changes were observed in the pancreas, impaired fasting glucose might result from the steatosis in liver. Generally, impaired fasting glucose is predictive for prediabetes, and the onsets of T1D and T2D, such as high fasting glucose and low insulin, seem to be similar, which was why few pathways were screened from short-term animal experiments. This discrepancy may result from the different exposure of environmental pollutants. Human are exposed to complex chemicals mixture including BDE47, while the animals are exposed to BDE47 alone. In addition, the exposure to BDE47 is low-dose and long-term in humans while it is short-term subchronic in animals. However, both styles contributed to the impaired fasting glucose in either humans or rats. So it is rational to understand that prolonged exposure to BDE47 might trigger the progression of prediabetic states to full-blown T2D.

This community-based case-control study and animal experiments as well as high throughput genomic analysis suggested BDE47 exposure predicted the future risk of diabetes. Findings must be confirmed by prospective studies with more strict diagnostic criteria for diabetes, such as glucose-tolerance test and HbA1c in other populations. In addition, more research efforts in animal studies in terms of long-term low doses of BDE47 treatment are needed to further reveal diabetogenic mechanisms. Nevertheless, results add to the existing evidence supporting policies to decrease human exposure to PBDEs, and other environmental pollutants as well. In addition to efforts for decreasing exposures, there is particular need of improved mechanism-based screening and testing tools for safety assessment of persistent chemicals with bioaccumulation potential.

## Methods

### Study population

The two community-based case-control studies were derived independently from two randomized cluster cross-sectional investigations, respectively. All the participants signed an informed consent before participation in the study. In the crude study I, 2715 subjects, including 1077 men and 1638 women, were enrolled in the mid Jiangsu province from March to May in 2011. In the crude study II, 6209 subjects, including 2412 men and 3797 women, were enrolled in the northern Jiangsu province from March to August in 2012. All the participants underwent a health examination and a face-to-face questionnaire including general information, medical history and the use of medications, family history of diseases, lifestyle, and so on. Two tubes of 5 mL venous blood samples were collected, and serum and plasma were separated immediately. The biochemical indices were then measured, including total cholesterol (TC), triglycerides (TG), high density lipoprotein cholesterol (HDLC), low density lipoprotein cholesterol (LDLC), alanine transarninase (ALT), aspartate aminotransferase (AST), blood urea nitrogen (BUN), creatinine (CREA), and blood uric acid. Participants were considered as diabetic if they were previously diagnosed in hospital, or their fasting plasma glucose was ≥7 mmol/L confirmed at least twice in different periods during the studies^31^, and was then validated by local hospitals. Thus, 245 cases and 245 sex and age matched controls were enrolled in study I, and 565 cases and 565 sex and age matched controls were enrolled in study II. The present study was approved by the Institutional Review Board of Nanjing Medical University, and conducted according to the principles expressed in the Declaration of Helsinki.

### Animal experiment

Animals and administration procedures were described in our previous study^32^. Briefly, forty adult male Sprague-Dawley (SD) rats (220–250 g) were randomly divided into four groups (10 rats per group), and they were orally administered 0.001, 0.03, and 1 mg/Kgd BDE47 (purity ≥98.7%; Chemservice, West Chester, PA, USA) dissolved in 0.1 ml corn oil for 8 weeks (6 consecutive days per week), and the control rats received a same volume of corn oil only. After the treatment fasting glucose was determined routinely by an autoanalyzer (Hitachi 7000, Japan) using standard kits from Jiancheng bio-engineering research institute (Nanjing, China). The serum insulin was determined by enzyme-linked immunosorbent assays (ELISA) kits obtained from Cusabio BIOTECH Co., Ltd (Wuhan, China). The care and use of the animals were followed the animal welfare guidelines, and all the experimental protocols were approved by the Animal Care and Welfare Committee of Nanjing Medical University.

### Detections of serum PBDEs and BDE47 in human and rats

Seven PBDE congeners including BDE28, BDE47, BDE99, BDE100, BDE153, BDE154, and BDE183 in human serum, and BDE47 in rat serum were measured. The sample extraction and clean-up for analysis was performed as described previously^32^,^33^. The samples were analyzed with a gas chromatograph-mass spectrometer (GC/MS) (Thermo Finnigan DSQ, USA) using BDE77 as an internal standard. Briefly, 1 μL of sample was injected using the splitless injection mode with a splitless time of 1 min. The mass spectrometer was operated under negative chemical ionization. Selected ion monitoring (SIM) was employed to determine an individual peak. The following pair of ions were monitoring for each target compound with the first one being used to quantify an ion and the second one for peak identification: *m/z* 81 and *m/z* 79. Representative GC/MS chromatograms of each PBDE from the calibration mix (Accustandard, New Haven, CT, USA), a serum sample, and the corresponding standard curve are shown in Fig. S3. The recovery, relative standard deviation (RSD), limit of detection (LOD), and maximum levels in human serum were listed in Table S4.

### Morphological observation of rat livers and pancreatic islets

The liver and pancreas of the rats were fixed in 4% neutral buffered formalin and dehydrated with graded ethanol. Each tissue was embedded in paraffin, and 5 μm cross-sections were prepared and stained with hematoxylin and eosin (H&E). The histopathological profiles were observed using an optical microscope (Axioskop 2 plus, Carl Zeiss, Hamburg, Germany).

### Microarray hybridization and data analysis

Three rats were selected randomly from the control and BDE47-treated group (0.03 mg/Kg). Liver RNA was extracted with the RNeasy Mini Kit (Qiagen, Valencia, CA, USA), and the quality of total RNA was evaluated by an A260/A280 ratio and electrophoresis on an Agilent Bioanalyzer. Total RNA was submitted to Peking University Stem Cell Research Center for sample processing and chip hybridization according to the manufacturer’s instructions. The arrays were scanned with the Affymetrix Gene Chip scanner 3000 and the signals were processed by GCOS 2.0 tools. GeneSpring software 9.0 (Agilent) was used to filter out the probesets (genes) with all “absent calls” (no detectable signals) among all arrays. The signal values of the remaining probesets were transformed to logarithm at base 2. The quality control of genechips and further analysis of gene ontology (GO) category, pathway, genes-act-network, and gene co-expression were executed as described in our previous study^13^.

### Statistical analysis

All statistical differences were analyzed using SPSS software (17.0) (Chicago, IL, USA). For human study, comparisons of general indicators between case and control groups were conducted by χ^2^ test using a univariate analysis model. Based on a significance level of *P* ≤ 0.10 as the selection of candidate factors, the individual influence on the prevalence of diabetes was analyzed using a multivariate logistic regression model. Wilcoxon signed rank test was used to assess the differences of PBDEs concentrations in diabetes. For each PBDE, participants with concentrations below the corresponding LOD were regarded as the reference group, and those with detectable values were categorized by cut points at the 1^st^ and 2^nd^ tertiles. Logistic regression models were used to calculate multivariable adjusted odd ratios (ORs) and 95% confidence intervals (CIs). For animal study, the results were represented as mean ± standard deviation (SD), the comparison among groups in rats was conducted using one-way analysis of variance. The differences were considered statistically significant at *P* < 0.05, either human study or animal study.

## Additional Information

**How to cite this article**: Zhang, Z. *et al*. Environmental exposure to BDE47 is associated with increased diabetes prevalence: Evidence from community-based case-control studies and an animal experiment. *Sci. Rep*. **6**, 27854; doi: 10.1038/srep27854 (2016).

## Supplementary Material

Supplementary Information

Supplementary Table S1

Supplementary Table S2

## Figures and Tables

**Table 1:** Distribution of typical characteristics in cases and controls.

Characteristics	Study I (N = 490)			Study II (N = 1130)		
	Control n (%)	Case n (%)	*P*	Control n (%)	Case n (%)	*P*
Current smoking	75 (30.6)	71 (29.0)	0.693	136 (24.1)	146 (25.8)	0.492
Current drinking	53 (21.6)	61 (24.9)	0.392	113 (20.0)	113 (20.0)	1.000
Family history of hypertension	32 (13.1)	37 (15.1)	0.516	67 (11.9)	70 (12.4)	0.785
Family history of diabetes	7 (2.9)	18 (7.3)	0.024	9 (1.6)	33 (5.8)	0.000
Hypertension	82 (33.5)	148 (60.4)	0.000	222 (39.3)	354 (62.7)	0.000
Hypercholesterolemia	11 (4.5)	40 (16.3)	0.000	19 (3.4)	89 (15.8)	0.000
Hypertriglyceridemia	16 (6.5)	72 (29.4)	0.000	21 (3.7)	165 (29.2)	0.000
HDLC < 1.40 mmol/L	94 (38.4)	124 (50.6)	0.006	141 (25.0)	212 (37.5)	0.000
LDLC > 4.14 mmol/L	11 (4.5)	29 (11.8)	0.003	11 (1.9)	74 (13.1)	0.000
Dyslipidemia	103 (42.0)	162 (66.1)	0.000	156 (27.6)	333 (58.9)	0.000
Central obesity	39 (15.9)	95 (38.8)	0.000	86 (15.2)	185 (32.7)	0.000
Obesity	23 (9.4)	57 (23.3)	0.000	45 (8.0)	128 (22.7)	0.000
ALT > 40 U/L	4 (1.6)	2 (0.8)	0.411	9 (1.6)	8 (1.4)	0.807
AST > 40 U/L	10 (4.1)	11 (4.5)	0.823	38 (6.7)	30 (5.3)	0.317
BUN > 7.14 mmol/L	34 (13.9)	34 (13.9)	1.000	92 (16.3)	105 (18.6)	0.308
Hyperuricemia	6 (2.4)	20 (8.2)	0.005	30 (5.3)	61 (10.8)	0.001
CREA > 133 μmol/L	4 (1.6)	3 (1.2)	0.703	39 (6.9)	46 (8.1)	0.430

**Table 2:** Multivariate logistic regression analysis of candidate factors related to diabetes.

	Study I (N = 490)			Study II (N = 1130)		
	OR	(95% CI)	*P*	OR	(95% CI)	*P*
Central obesity	3.792	(1.939, 7.418)	0.000	2.625	(1.699, 4.055)	0.000
Obesity	2.209	(1.120, 4.357)	0.022	2.107	(1.305, 3.403)	0.002
Hypertension	2.700	(1.648, 4.424)	0.000	2.060	(1.483, 2.863)	0.000
Dyslipidemia	3.296	(1.882, 5.774)	0.000	4.506	(3.089, 6.574)	0.000
Family history of diabetes				3.873	(1.606, 9.337)	0.003

**Table 3:** Comparison of detection rates and median concentrations of PBDEs between cases and controls.

		Study I (N = 490)			Study II (N = 1130)		
PBDEs		Control	Case	*P*	Control	Case	*P*
BDE28	Detection rates (%)	71.0	54.3	0.000	38.9	42.7	0.204
	Median concentration (pg/ml)	44.9	63.6	0.147	48.4	46.0	0.236
BDE47	Detection rates (%)	45.7	58.0	0.007	34.3	45.1	0.000
	Median concentration (pg/ml)	19.0	31.0	0.019	20.6	24.0	0.000
BDE99	Detection rates (%)	1.2	1.2	1.000	5.3	3.2	0.077
	Median concentration (pg/ml)	25.0	16.0	0.700	69.0	19.0	0.074
BDE100	Detection rates (%)	10.2	7.3	0.264	7.1	5.8	0.397
	Median concentration (pg/ml)	9.4	7.2	0.205	17.2	15.0	0.391
BDE153	Detection rates (%)	6.5	5.7	0.706	12.4	11.3	0.581
	Median concentration (pg/ml)	8.6	7.3	0.101	8.8	13.9	0.629
BDE154	Detection rates (%)	29.4	33.5	0.330	19.6	23.0	0.168
	Median concentration (pg/ml)	16.0	15.9	0.954	18.8	15.0	0.295
BDE183	Detection rates (%)	41.2	39.6	0.713	31.3	33.1	0.524
	Median concentration (pg/ml)	26.5	30.5	0.890	41.5	35.5	0.625

The median concentrations of PBDEs were calculated with detectable values.

**Table 4:** Adjusted ORs and 95% CI of prevalent diabetes categorized by BDE47.

Study		Not Detectable	Detectable		*P*_*trend*_
			1st tertile	2st tertile	
Study I	Case/n	103/236	60/127	82/127	0.001
	Adjusted OR (95% CI)	Reference	1.13 (0.71, 1.82)	2.10 (1.29, 3.40)	0.009^a^
Study II	Case/n	310/681	120/224	135/225	<0.001
	Adjusted OR (95% CI)	Reference	1.15 (0.83,1.61)	1.68 (1.20, 2.36)	0.010^b^

^a^Adjusted for hypertension, dyslipidemia, and central obesity.

^b^Adjusted for hypertension, family history of diabetes, dyslipidemia, central obesity and obesity.
