# Supplementary material for: Environmental exposure to BDE47 is associated with increased diabetes prevalence: Evidence from community-based case-control studies and an animal experiment
Source: Sci Rep. 2016 Jun 13;6:27854. doi: 10.1038/srep27854 (PMC4904204; doi:10.1038/srep27854)
Supplement: Supplementary Information [file srep27854-s1.pdf]

Environmental exposure to BDE47 is associated with increased diabetes prevalence: Evidence from community-based case-control studies and an animal experiment

Zhan Zhang, Shushu Li, Lu Liu, Li Wang, Xue Xiao, Zhenzhen Sun, Xichen Wang, Chao Wang, Meilin Wang, Lei Li, Qiujin Xu, Weimin Gao, Shou-Lin Wang

Supplementary Information

Methods

Analysis of real time quantitative reverse transcription PCR (qRT-PCR). qRT-PCR was performed to quantify gene expression to validate the data obtained from microarray analysis. Three rats were selected randomly from the control and BDE47-treated group (0.03 mg/Kg) to extract RNA. Total RNA were reverse transcribed using Oligo(dT)18 Primer (Promega, Madison, WI, USA) in the presence of Omniscript Reverse Transcriptase (Qiagen, Valencia, CA, USA). qRT-PCR was performed using FastStart Universal SYBR Green Master (Roche, Mannheim, Germany) on a 7300 Fast Real Time PCR System (Applied Biosystems, Life Technologies Corporation, Warrington, UK) per manufacturer's instructions. All samples were run in triplicate, and the relative gene expression was analyzed according to the $2^{-\Delta\Delta Ct}$ method. The housekeeping gene, glyceraldehyde phosphate dehydrogenase (GAPDH), was used to normalize the expression data.

Definition of human health examination. During the health examination, the participants' weight and height in standing position without shoes were measured. The body

mass index (BMI; Kg/m^2) was calculated from data on height (m) and weight (Kg) collected at the health examination. Two different cut off values were used: 24 ≤ BMI < 30 (overweight) and BMI ≥ 30 (obesity). The hip and waist circumferences (from the high point of the iliac crest to the nearest 0.1 cm at the end of normal expiration) were also measured. Central obesity was defined as the ratio of hip circumferences/waist circumferences > 1.0 in male. Blood pressure was also measured to evaluate the high blood pressure status, and the average of the last two measurements of blood pressure was used. Hypertension was defined as systolic pressure ≥ 140 mmHg or diastolic pressure ≥ 90 mmHg. Current smoking was defined as having smoked more than 1 cigarette per day for more than one year until now. Current drinking was defined as alcohol intake more than three times per week during the past 6 months.

Some of the parameters measured in serum were total cholesterol (TC), triglycerides (TG), high density lipoprotein cholesterol (HDLC), low density lipoprotein cholesterol (LDLC), alanine transarninase (ALT), aspartate aminotransferase (AST), blood urea nitrogen (BUN), creatinine (CREA), and blood uric acid. Hyperuricemia was defined as the concentration of blood uric acid > 7.14 mmol/L. Hypercholesterolemia was defined as TC > 5.72 mmol/L. Hypertriglyceridemia was defined as TG > 1.70 mmol/L. The definition of dyslipidemia required that the participants satisfied two or more of the following four criteria: 1) TC > 5.72 mmol/L; 2) TG > 1.70 mmol/L; 3) HDLC < 1.4 mmol/L; and 4) LDLC > 4.14 mmol/L.

Results

Table S1 List of differentially expressed genes between BDE47 treatment and control groups. Folder change (FC), the relative expression level of genes in BDE47 treatment group compared with the control.

Table S2 Gene ontology (GO) categories identified using KEGG between BDE47 treatment and control groups. The top rank of GO categories identified by KEGG is listed according to *P*-values or enrichment.

Table S3. Verification of gene expression changes by qRT-PCR

Gene Name	Expression fold-change		Sequence of primers used in qRT-PCR
	Gene chip	qRT-PCR	
CYP7A1	0.40	0.31	Forward: TTCTCAATGACACGCTCTC<br>Reverse: GCTCCACTCACTTCTTCAG
CYP4A8	1.98	2.12	Forward: ACAGGCGATGGTTACTCAG<br>Reverse: CAGGGTCATACACTTGGATTC
UDPG2B	1.90	2.01	Forward: GAAGTACGTCACTGGACTAAGC<br>Reverse: ATGTAGAGTAGCGGAGAAGGC
CYP2B15	1.97	1.30	Forward: GGAAGATGTGAGCAGTGG<br>Reverse: CAAGGAGAGTGGCATTGG
Adipoq	5.13	2.67	Forward: TGTTCCTCTTAATCCTGCCCAGT<br>Reverse: GTCTCCCTTCTCTCCCTTCTCTC
Tnf	3.61	1.28	Forward: AGCAAACCACCAAGCGGAGG<br>Reverse: CAGCCTTGTCCCTTGAAGAGAAC
Prkaa1	3.27	1.45	Forward: TAAACCCACAGAAATCCAAACACC<br>Reverse: ACAACCTTCCATTCATAGTCCAACT
Glp1r	0.15	0.23	Forward: GAGTCCAAGCAAGGAGAGAGAAA<br>Reverse: TGACCAAGGCAGAGAAAGAAAGT
Ednra	3.76	4.15	Forward: AGTGGAAGAACCAGGAGCAGAAC<br>Reverse: GACAAAAAGCAGGGGAGAGACC
Ins2	4.08	16.53	Forward: GGAAACCATCAGCAAGCAGGTC<br>Reverse: AAGAATCCACGCTCCCCACAC
Drd1a	0.19	0.64	Forward: TGTTTGTGTGGTTTGGGTGG<br>Reverse: TATGGCATTATTCGTAGTAGGGC

Table S4. Detection profiles of PBDEs in the participants' serum

PBDEs	Recovery (%)	RSD (%)	LOD (pg/mL)	Maximum (ng/mL)
BDE28	84.7	9.0	0.91	0.5339
BDE47	96.2	3.6	0.70	4.6398
BDE99	91.0	0.9	0.97	0.0871
BDE100	90.2	1.8	1.00	0.1053
BDE153	89.5	6.4	0.88	0.1505
BDE154	98.7	4.7	0.81	0.8760
BDE183	93.0	1.2	1.36	1.3666

RSD, relative standard deviation; LOD, limit of detection.

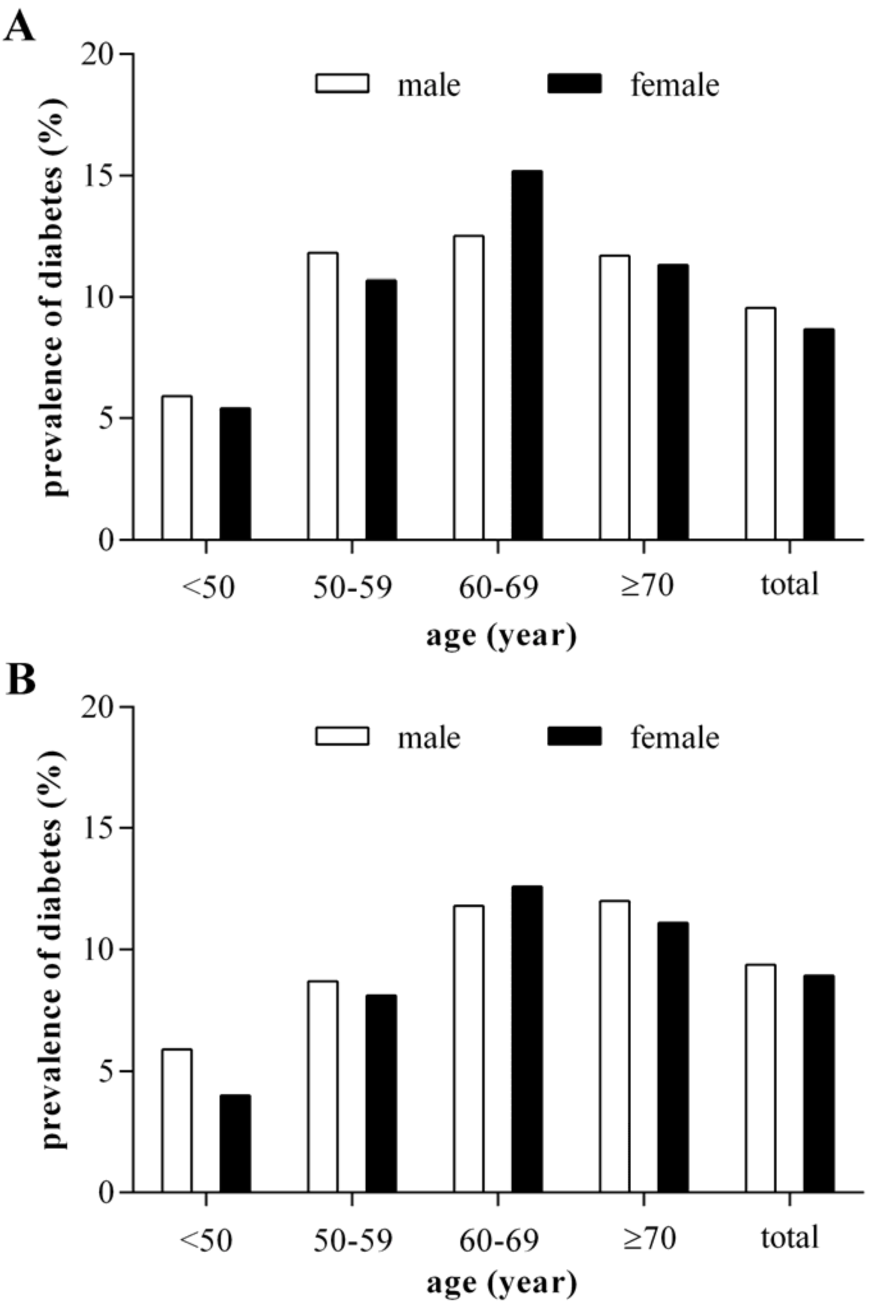

Figure S1 Age-specific prevalences of diabetes. (A) The prevalences of diabetes among men and women in study I. (B) The prevalences of diabetes among men and women in study II.

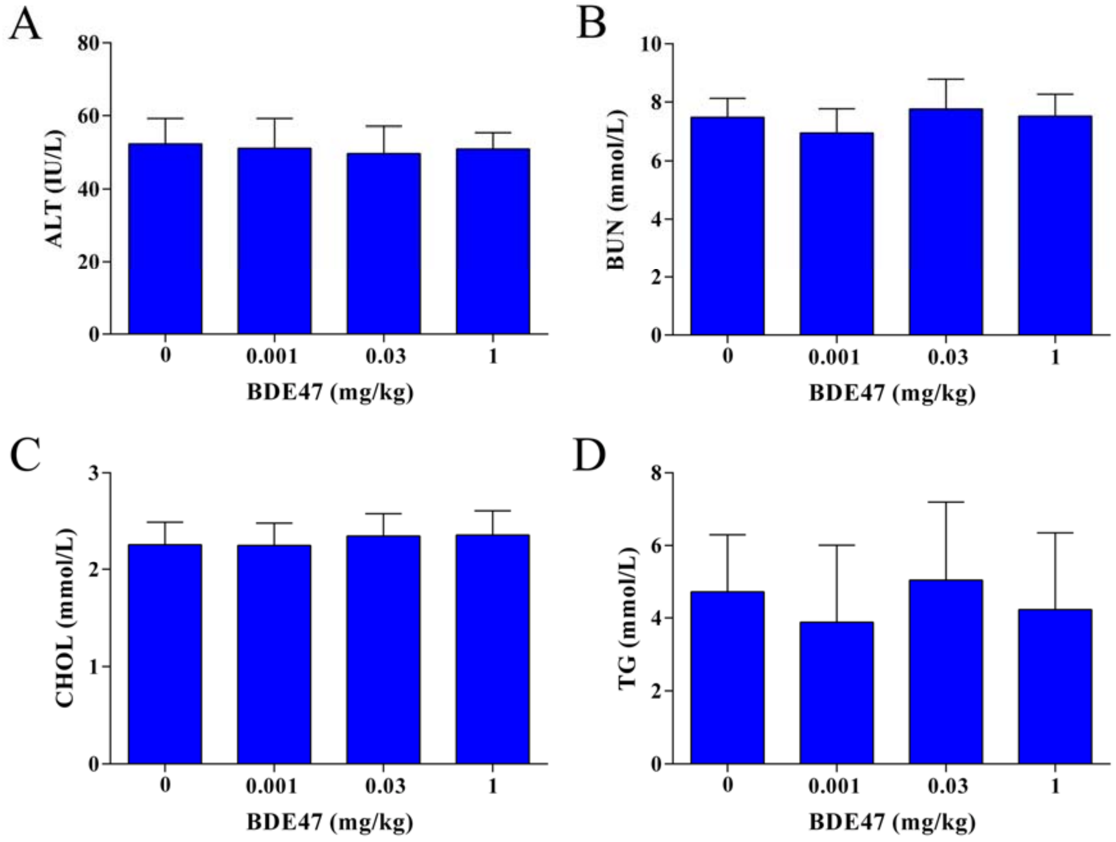

Figure S2 Effects of BDE47 on serum biochemical parameters. Plasma antioxidant activity such as (A) alanine transarninase (ALT), (B) blood urea nitrogen (BUN), (C) cholesterol (CHOL), and (D) triglyeride (TG) were determined after 8 weeks of BDE47 exposure. The results are expressed as the mean ± SD of 10 rats in each group.

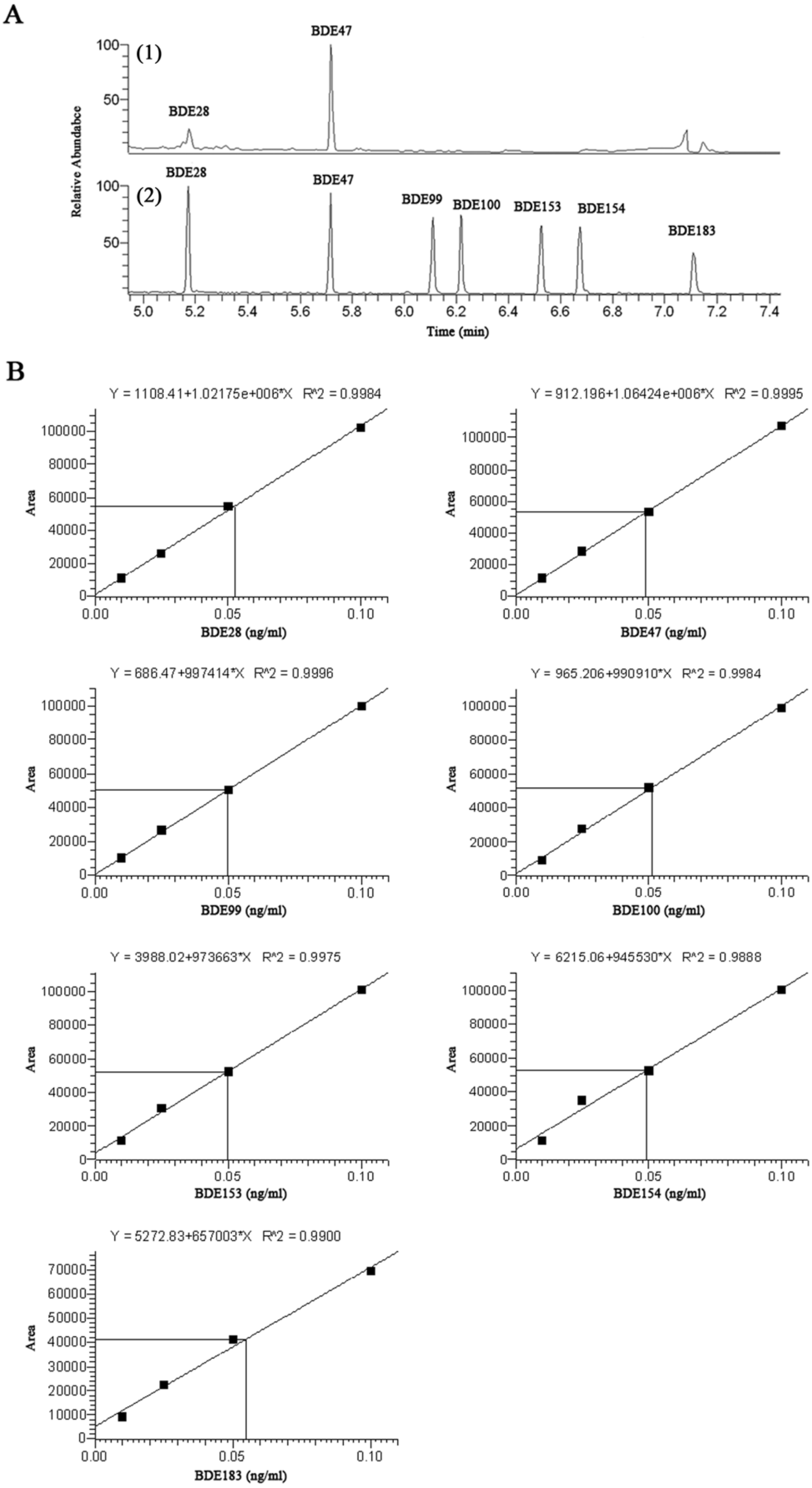

Figure S3 Detection of PBDEs. (A) GC/MS chromatograms of representative PBDE congeners (upper) and human serum sample (below), (B) the corresponding standard curve**.**
